# Phylogeographic Analysis for Understanding Origin, Speciation, and Biogeographic Expansion of Invasive Asian Hornet, *Vespa velutina* Lepeletier, 1836 (Hymenoptera, Vespidae)

**DOI:** 10.3390/life14101293

**Published:** 2024-10-12

**Authors:** Xuhua Xia

**Affiliations:** 1 Department of Biology, University of Ottawa, Ottawa, ON K1N 9A7, Canada; xxia@uottawa.ca; 2 Ottawa Institute of Systems Biology, University of Ottawa, Ottawa, ON K1H 8M5, Canada

**Keywords:** conservation biology, DNA barcoding, geophylogeny, index of invasiveness, invasive species, phylogeography, speciation, *Vespa velutina*, yellow-legged hornet

## Abstract

The Asian hornet, *Vespa velutina*, is an invasive species that has not only expanded its range in Asia but has also invaded European countries, and it incurs significant costs on local apiculture. This phylogeographic study aims to trace the evolutionary trajectory of *V. velutina* and its close relatives; it aims to identify features that characterize an invasive species. The last successful invasion of *Vespa velutina* into France occurred in late May, 2002, and into South Korea in early October, 2002, which were estimated by fitting a logistic equation to the number of observations over time. The instantaneous rate of increase is 1.3667 for *V. velutina* in France and 0.2812 in South Korea, which are consistent with the interpretation of little competition in France and strong competition from local hornet species in South Korea. The invasive potential of two sister lineages can be compared by their distribution area when proper statistical adjustments are made to account for differences in sample size. *V. velutina* has a greater invasive potential than its sister lineage. The ancestor of *V. velutina* split into two lineages, one found in Indonesia/Malaysia and the other colonizing the Asian continent. The second lineage split into a sedentary clade inhabiting Pakistan and India and an invasive lineage colonizing much of Southeast Asia. This latter lineage gave rise to the subspecies *V. v. nigrithorax*, which invaded France, South Korea, and Japan. My software PGT version 1.5, which generates geophylogenies and computes geographic areas for individual taxa, is useful for understanding biogeography in general and invasive species in particular. I discussed the conceptual formulation of an index of invasiveness for a comparison between sister lineages.

## 1. Introduction

The genus *Vespa*, natively distributed in tropical, subtropical, and temperate Asia, features several invasive species such as *V. crabro* Linnaeus 1758 and *V. velutina* Lepeletier, 1836 (Hymenoptera, Vespidae) [1,2,3] and potentially invasive species such as *V. mandarinia* Smith, 1852 [4,5,6,7]. Several hornet species are mass murderers of honeybees [8,9], and the diversity and distribution of hornets closely mirror those of honeybees. This dependence of the hornets on honeybees for food led to an interesting evolutionary twist. An orchid species, *Dendrobium sinense*, has evolved a function of emitting a honeybee pheromone to attract hornets for pollination [10].

*Vespa velutina* is the most notorious invasive species among hornets. Its invasion of Europe [11] severely affected European apiculture, leading to tens of millions of dollars in management costs [12]. While the Asian honeybee (*Apis cerana*) has evolved a special thermal defense against local hornets [13,14], the European honeybee (*Apis mellifera*) remains largely defenseless against this new predator [9,15,16]. A defense by the balling behavior has been observed in Cyprian honeybees, *A. mellifera cypria*, against the sympatric oriental hornets, *V. orientalis* [17], and in Italian honeybees, *A. mellifera ligustica,* against the sympatric European hornet, *V. crabro* [18]. However, the defensive behavior of *A. mellifera ligustica* is not effective against *V. velutina* [18]. Probably because of the generally weaker defense in *A. mellifera* than in *A. cerana* against hornet predators, the bee-hawking success rate is three times greater when *V. velutina* prey on *A. mellifera* than on *A. cerana* [19].

There are 22 morphologically distinguishable hornet species in the genus *Vespa* living in Southeastern Asia [20,21,22], but why does *V. velutina* appear more invasive than others? Of the 11 to 13 recognized subspecies [22,23,24], why was only the subspecies *V. velutina nigrithorax* du Buysson, 1905 successful in Europe, South Korea, and Japan [25,26,27,28]? Is it an accidental introduction that could have happened to any other hornet species or subspecies, or is it because *V. v. nigrithorax* possesses features that are more conducive to colonization? What features are required for a successful invasive species?

A successful invasive hornet would need to overcome at least seven obstacles to start a new population in a new habitat. First, the mated queen (known as foundress) needs to have a means of long-distance dispersal, either by self-propelled flight or by hitchhiking. Second, the queen needs to find a nest site and build its first nest. Third, she and her offspring must find suitable food. Fourth, the colony has to grow rapidly to avoid accidental loss of the colony. Fifth, because the foundresses are likely few or even just one, at least some of her fertilized eggs should be relatively free of deleterious recessive alleles so that some of her descendants will not suffer from inbreeding depression. Sixth, the colony needs to neutralize new hymenopteran pathogens [29] and escape new predators such as the European honey buzzard [30] and the European bee-eater, *Merops apiaster* [31]. Seventh, the newly produced queens need to find mates that are relatively free of deleterious recessive alleles to propagate the colonies into future generations.

**Figure 1:**
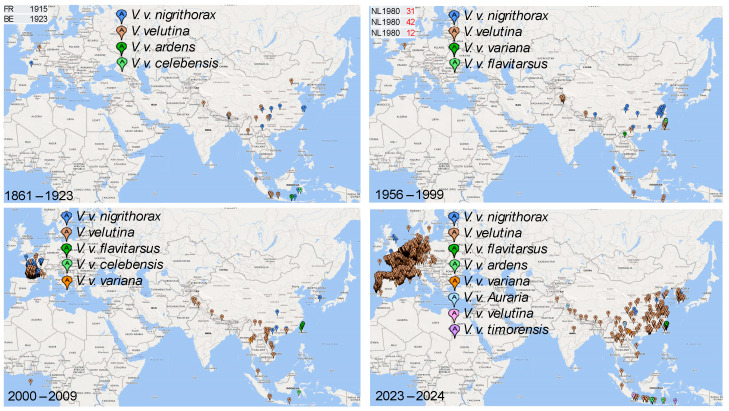
Recorded sightings of *V. velutina* in the Old World showing the expansion of *V. velutina* in Europe. The time period for each of the four sub-figures is in the lower left. The three European countries (FR, BE and NL for France, Belgium and The Netherlands) and the year when *V. velutina* sitings were recorded are in the top-left of the two upper sub-figures. The map marker indicates the subspecies within *V. velutina.* All *V. velutina* specimens in Europe belong to *V. velutina nigrithorax,* but some do not have the subspecies designation. Thus, at least for the European specimens, the two map markers, one for *V. v. nigrithorax* and the other for *V. velutina,* actually designate the same *V. velutina nigrithorax* subspecies.

Although hornets are not as strong fliers as locusts or katydids, which can fly across the Pacific to colonize Hawaiian Islands [32], they are known to hitchhike over long distances, which must be how *V. mandarinia* foundresses reached North America from Asia [5,6,16,33]. Foundresses of *Vespa velutina* tend to choose small and dark cavities to hibernate [34]. They can, therefore, be transported long distances while hibernating. Hitchhiking to Europe must have occurred multiple times for *V. velutina*; this is based on the observation recorded in the GBIF database [35]. *V. velutina* was first observed in France and Belgium in 1915 and 1923, respectively (Figure 1), but then must have disappeared. It was again observed in the Netherlands in 1980 (Figure 1) but again, it disappeared. It was also observed twice in 2003 and four times in 2004, according to GBIF records [35], but only the observation in 2005 was described in detail and published [36,37,38] with the conclusion that *V. velutina* was already well established in several counties in France. One should not confuse these locally collected specimens with those specimens collected elsewhere but stored in local museums. For example, the Natural History Museum stores a *V. velutina* specimen (NHMUK010636232) collected in Sumatra in 1914, which should not be misinterpreted as an observation of *V. velutina* in the UK in 1914.

**Figure 2:**
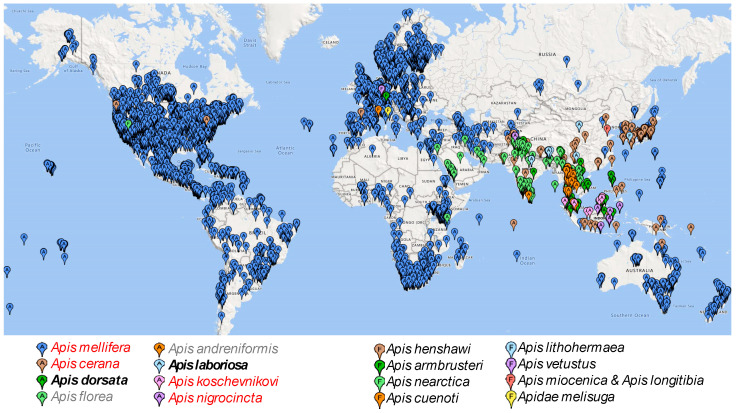
Geographic distribution of honeybee species. Map markers with letter A indicate alive/extant, whereas those with letter F indicate fossils.

While food is often a key environmental factor limiting the geographic distribution of many species, it is not so in the case of hornets. *V. velutina* prey mostly on honeybees [25], which contribute two-thirds of the diet of *V. velutina* in an urban environment and about one-third in farmland and forests [25]. The global distribution of honeybees (Figure 2) shows the availability of food for hornets. The highest species diversity of honeybees is observed in Southeastern Asia (Figure 2), which corresponds to the subspecies richness in *V. velutina* (Figure 1).

After *V. velutina* was observed on 1 November 2005 [36,37], the invasive species quickly expanded its range in France and from France to other European countries. Where is the source population from which the first founding queen initiated the successful colonization? When did the foundresses of the last successful colonization arrive in France? Is it much earlier than 2005? How rapidly can the population size increase over the years? These can be addressed by estimating the initial invasion time and the instantaneous rate of growth.

The source population is often identified by using genetic data such as DNA barcoding data [39] when two requirements are met [40]. To identify which *V. velutina* population in Southeast Asia is the source population for the European *V. velutina*, one first needs to characterize all genetically unique populations in Southeast Asia and then identify which *V. velutina* population in Southeast Asia is genetically identical or the most closely related to the European *V. velutina.* If all individuals of *V. velutina* in Southeast Asia were genetically identical, then any *V. velutina* population in Southeast Asia could be the source population. In contrast, if a population in northern Jiangsu province in China is genetically identical or the most similar to the European *V. velutina,* and if this population is also genetically unique in Southeast Asia, then this population is the best candidate as the source population for the European *V. velutina.* This approach has been used to identify the source population for *V. mandarinia* individuals found in Washington State in the US and British Columbia in Canada, with the source population in South Korea and Japan, respectively [16,33].

## 2. Material and Methods

Invasion time ($t_{0}$) and instantaneous rate of growth (*r*): The population size of one or more founding individuals will increase in size after the first colonization. If the population size is monitored over time long enough with fixed trapping stations, then this change in population size over time can be modeled by a logistic equation so that $t_{0}$ and *r* can be estimated:

(1) $$N_{t}=\frac{N_{0}K}{N_{0}+\left(K-N_{0}\right)e^{-r\left(t-t_{0}\right)}}$$

The logistic equation has four parameters: *K* (carrying capacity), *N*_0_ (initial population size), *r* (instantaneous rate of growth), and *t_0_* (the time of invasion). *N_t_* and *t* are dependent and independent variables, respectively. I provide the following justification for using the number of observations of *V. velutina* over the years as a proxy for *N_t_.*

The French government has sponsored insect-monitoring programs to create and update a complete insect inventory ever since the 19th century, especially during the Napoleonic period when the British imposed naval blockade so that France was not able to import insect-derived products such as cochineal from the Americas [41]. The number of reported observations (*n*) of *V. velutina* over the years might be proportional to population size, i.e., $n_{t}=cN_{t}$, where *c* is assumed to be constant, and can be used to estimate the four parameters: *K*, *N*_0_, $t_{0}$ and *r*. The resulting *K* and *N_0_* are not meaningful because we do not know the constant *c*, but this does not affect the estimation of *r* and *t_0_.* The number of observations of *V. velutina* in France over the years was downloaded from the Global Biodiversity Information Facility (GBIF) [35]. The GBIF records differ in reliability, many with specimens, many with photos, and some with only reported sightings (which should be the least reliable). However, excluding the last category does not change the conclusions in this paper.

The assumption of a constant *c* above could be problematic. If a country initiates a large-scale effort to monitor insects in year *T*, then the number of observations of insects after *T* would be greater than that before *T* simply because of the change in observation effort, not necessarily because of the change in population size. There are also many other factors that could affect observation efforts, e.g., wars. In the French case, there are no known significant changes in observation effort, so the assumption of a constant *c* might be reasonable. However, the spread of *V. velutina* in France and in other European countries, as well as the detrimental effect on apiary, has resulted in a large-scale coordinated effort to eliminate the invasive hornet in 2012. This is legitimized by the French government’s classification of *V. velutina* as an invasive alien species harmful to beekeeping in response to the repeated requests from beekeepers’ associations for several years. This classification allowed the development and implementation of mandatory control programs at the national and departmental levels. Although the elimination effort failed, the population size of *V. velutina* decreased dramatically, so I included only observation data up to 2011.

DNA sequence data and geographic coordinates of sample sites: I downloaded the mitochondrial sequences from GenBank and the Barcode of Life Data (BOLD) System [39]. One of the sequences annotated as from *V. velutina* (JQ780459) must have resulted from a species misidentification because the sequence is nearly identical to the COI sequence in *V. affinis* but very different from all known *V. velutina.* This sequence is excluded. I wish to highlight this partly because the sequence has been used in other studies [42,43] as a *V. velutina* sequence and partly because of the many misannotations in GenBank sequences [44]. For specimens with complete mitochondrial genomes, the COX1 sequences were extracted from the GenBank file with DAMBE [45]. COX1 sequences from *Vespa simillima* Smith, 1868, *Vespa bicolor* Fabricius, 1787 and *Vespa vivax* Smith, 1870 were used as outgroups. Previous studies [46,47,48,49] have shown that *V. simillima*, *V. bicolor* and *V. vivax* are closely related to *V. velutina. V. simillima* appears to occupy an ecological niche similar to *V. velutina* because the invasion of *V. velutina* into South Korea displaces the native *V. simillima* [28].

Geographic coordinates were mostly from the BOLD System. However, the BOLD System does not include proper geographic coordinates for some specimens but instead, lists the center of the country as the geographic coordinates. These are obviously unsatisfactory and were replaced by approximate geographic coordinates of the sampling location recorded in the original publication. Several sequences in China have only provincial information. Sequences JQ780449_Zhejiang1 and JQ780451_Zhejiang2 were assigned the average city coordinates of Zhejiang Province but with a slight difference in longitude to avoid complete overlap when plotting the geophylogeny onto the map. Sequence JQ780450_Jiangsu/Zhejiang was assigned the geographic coordinates of the point in the middle of the provincial borders. The sequence identification (GenBank accession and BOLD System ID) for downloaded sequences, the latitude and longitude, and GC% were included in Table S1 in the Supplemental file.

The resulting *COX1* sequences were aligned using MAFFT [50] version 7 with the most accurate LINSI option (‘-localpair’ and ‘-maxiterate = 1000’). For phylogenetic reconstruction with PhyML [51] Version 3, the GTR + Γ model was used with four discrete rate categories for approximating a continuous gamma distribution [52]. This model was chosen based on the information-theoretic index AIC and the likelihood ratio tests [53,54] among the nested HKY [55], TN93 [56], and GTR [57,58] models with or without the discrete gamma distribution to accommodate rate heterogeneity in substitution rate among sites. However, the TN93 + Γ model generated the same tree with negligible difference in branch lengths. The tree improvement option (‘-s’) was set to ‘BEST’ (best of NNI and SPR search). The ‘-o’ option was set to ‘tlr’, which optimizes the topology, the branch lengths, and rate parameters. MAFFT and PhyML are included in DAMBE and called to analyze sequences with a consistent user interface.

For sister lineages that have evolved over the same amount of time, a sister lineage with a wider geographic distribution than the other indicates that the former is likely more invasive than the latter. To measure the area of geographic distribution of a taxon, I used the convex hull algorithm [59] implemented in the PGT software version 1.5 [60]. Approaches to alleviate the confounding factors were taken and discussed in the result interpretation.

Geophylogenies were produced with PGT [60], which takes two types of information: a phylogeny and a list of specimens with associated latitude and longitude. When specimens from two subspecies share exactly the same latitude and longitude, the map marker of one specimen would completely cover the other. For this reason, their geographic coordinates are slightly shifted from each other to avoid complete overlap. The objective of a geophylogeny is to visualize phylogenetic relationships and biogeographic distribution of evolutionary lineages [32,33]. We used PGT software version 1.5 [60] to generate geophylogenies for visualization. PGT makes use of both Google Maps and Microsoft Bing Maps, which have regular map views and satellite terrain views.

## 3. Results

### 3.1. Estimating Invasion Time ($t_{0}$) and Instantaneous Rate of Growth (r)

A logistic equation was fitted to the number of observations of *V. velutina* in France over the years, which were downloaded from GBIF [35]. Only data from 2003 to 2011 were used because The French government officially classified *V. velutina* as an invasive pet to facilitate the implementation of mandatory control programs at the national and departmental levels [61]. An independent invasion event has happened in South Korea by the same subspecies, *V. v. nigrithorax* [28], with the first observation recorded in 2005 [35]. It has since increased in number and displaced native hornet species with similar ecological requirements and life history straits, such as *V. simillima* [28]. I estimated $t_{0}$ and *r* for both.

**Figure 3:**
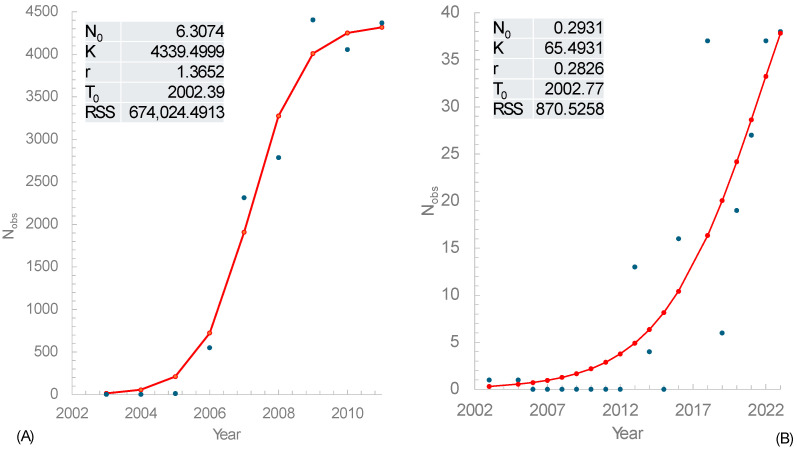
Estimating the timing of the last successful invasion event $t_{0}$, and *r,* the instantaneous rate of population growth of the invasive *V. velutina* in France (**A**) and South Korea (**B**). *N_obs_* is the number of observations of *V. velutina* recorded in GBIF [35]. RSS is the residual sum of squares in the least-squares estimation of parameters. The data from South Korea are supplemented with the observation of a *V. velutina* nest in 2003 [62], but the 14 specimens from the nest are counted as one observation. The blue and red dots are the observed values and their corresponding expectations, respectively.

As I have explained in the Methods and Materials, if we take the risk of assuming that the number of observations (*N_obs_*) of *V. velutina* recorded in GBIF [35] is proportional to population size (*N_t_*), then we can estimate the four parameters in Equation (1), i.e., $N_{0},K,\;t_{0}$ and *r*, where $t_{0}$ is the time of the last successful invasion, and *r* is the instantaneous rate of population growth. The four parameters were estimated by the least-squares method (Figure 3). $t_{0}$ was in early 2002.40, and r=1.3667, in France (Figure 3A). The corresponding values for South Korea are 2002.77 and 0.2812, respectively (Figure 3B). Because we assume that $N_{obs}=cN_{t}$ but we do not know the constant *c*, the values of $N_{0}$ and K in Figure 3 are not meaningful because the true $N_{0}$ and K should be multiplied by the constant *c* that we do not know.

Two points are worth making. First, *N_obs_* is much greater for France than for South Korea, so the observed data points are much closer to the fitted curve in the population in France than that in South Korea. Second, the estimated *r* is much smaller for the population in South Korea than in France, which is consistent with the following interpretation. There are at least seven *Vespa* species and three subspecies in South Korea, as reported in 1994 [27], so the ecological niche for *Vespa* species is perhaps already crowded [42], although it is not clear if they exhibit microhabitat separation. This is consistent with the observation that the expansion of *V. velutina* in South Korea is associated with the displacement of the native *V. simillima* [28]. This interspecific competition would slow down the growth in population size for *V. velutina* as a new immigrant. In contrast, there is only one native hornet species, *V. crabro,* in France, and *V. velutina* and *V. crabro* do not affect each other negatively [63], suggesting possible niche divergence between the two species. However, given that both *V. velutina* and *V. crabro* prey upon bees, some competition between the two is expected. Previous studies observed a slower spread of *V. velutina* in France [64] than in South Korea [28], consistent with the interpretation that *V. velutina* has encountered less competition in France than in South Korea.

I should emphasize the potential problems with the estimated parameters in Figure 3. First, the assumption of $N_{obs}=cN$, where N is the population size, may not be true as one could list multiple factors that could lead to a violation of the assumption. Second, even if the assumption is roughly true, the estimation in Figure 3B could be unreliable because of the small N_obs_ values as well as 0 observations in seven consecutive years (Figure 3B).

### 3.2. Phylogeographic Analysis

A phylogeographic analysis is fundamental for understanding invasive species. When two sister species (X and Y) diverged from a common ancestor, they would spread to their suitable habitats. After the same amount of divergence time, if species X gains a much wider geographic distribution than species Y, we may infer that species X has greater invasiveness than species Y.

**Figure 4:**
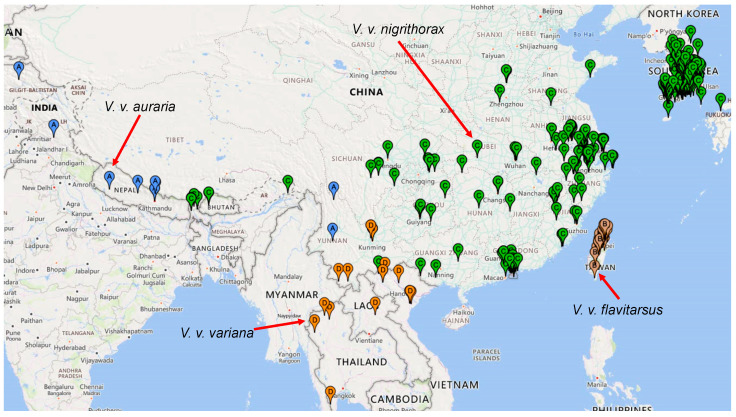
Sample distribution of the four most-sampled subspecies in *V. velutina,* based on data from GBIF [35]. The specimens from the same subspecies share the same map marker. Red arrows link the subspecies names to their map markers.

*Vespa velutina nigrithorax* is the subspecies that invaded Europe [36,37] and South Korea [62], and it features the widest distribution among all *V. velutina* subspecies [23] even without counting the area it recently colonized in Europe. This is also clear from the distribution of the four most sampled subspecies (Figure 4). However, it would be wrong to therefore conclude that *V. v. nigrithorax* is the most invasive. There are two confounding factors. The first is the timing of the subspecies recognition, which could be controversial [21]. If a subspecies were only recognized yesterday, then its samples would be few, and its distribution represented by its small samples would necessarily be much more limited than a subspecies that has been recognized and well-sampled for a hundred years. This problem can be overcome by using sampling points after the subspecies of interest have all been recognized. The other confounding factor is the divergence time. If subspecies X originated one million years ago and subspecies Y originated only 1000 years ago, then subspecies X is expected to have a wider distribution than species Y, even if they are equally invasive. Thus, valid comparisons in their invasive potential can only be made between sister lineages that have diverged for the same amount of time. For these reasons, phylogenetic analyses delineating sister lineages are essential for understanding invasive species.

**Figure 5:**
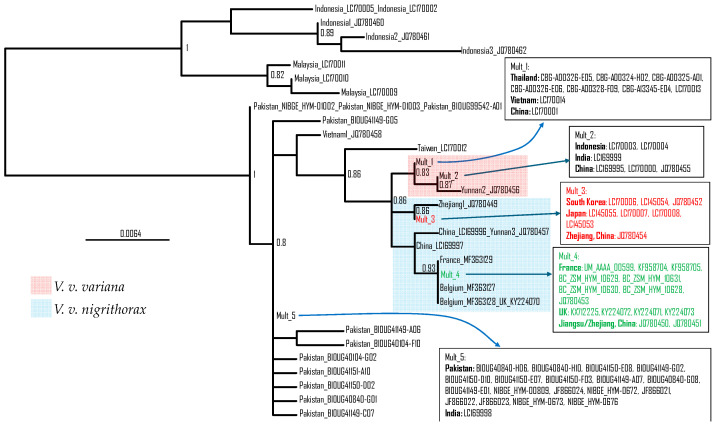
Phylogeny of *Vespa velutina*, mid-point rooted. Identical sequences are represented by Mult_#. The numbers next to internal nodes are bootstrap support values, with 1 meaning 100%. Leaf IDs are in the format of ‘Country_SequenceID’, where SequenceID are mostly GenBank accession, but some are BOLD System IDs.

The phylogenetic tree (Figure 5) is consistent with previous studies [42,43]. All specimens from South Korea and Japan share the same haplotype with a specimen from Zhejiang, China (JQ780454). These specimens are represented as Mult_3 in Figure 5 (colored red), and they are clustered with a specimen (JQ780449) collected in Zhejiang, China (Figure 5). It is reasonable to infer that the source population of the invasive individuals that colonized South Korea and Japan are from Zhengjiang, China. All specimens from Europe share the same haplotype as two specimens from Jiangsu and Zhejiang in China (JQ780450 and JQ780451) (Figure 5). Their only difference is at the two ends of the sequences because some sequences are longer than others. Therefore, the source population of the invasive individuals that colonized Europe was also from Zhejiang and Jiangsu provinces in China. The colonization of Europe and South Korea/Japan is by different lineages (Figure 5), i.e., by independent colonization events. The *V. velutina* found in South Korea and Japan are geographically close and located at two locations connected by high volumes of transportation, so the *V. velutina* in South Korea may be the secondary source population for those established in Japan because *V. velutina* was first found in South Korea and then in Japan [43].

The sister subspecies of *V. v. nigrithorax,* shaded blue (Figure 5), is *V. v. variana*, shaded pink (Figure 5). Visual inspection of Figure 4 suggests that *V. v. nigrithorax* has a wider distribution than *V. v. variana.* To obtain a quantitative measure of geographic distribution for comparison, I have computed the area of geographic distribution by the convex hull algorithm [59] implemented in PGT [60]. In calculating the area for *V. v. nigrithorax,* I excluded samples from both Europe as well as those from Japan and South Korea because these also resulted from recent invasion events in South Korea [27,28] and Japan [26,43,65,66]. The resulting area is 360.5129 square degrees (because the input is latitude and longitude in degrees) for *V. v. nigrithorax* and 47.1508 for *V. v. variana,* consistent with the interpretation that *V. v. nigrithorax* tends to be more invasive than *V. v. variana.*

**Figure 6:**
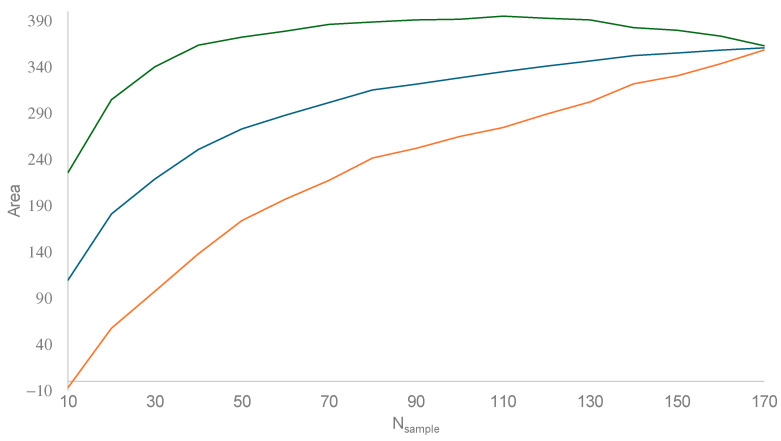
Subsampling the 170 sample points for *V. v. nigrithorax* (excluding sampling points in Europe and in Japan/South Korea). N_sample_ is the number of sample points randomly taken from the 170 sample points. Subsampling was repeated 100 times for each N_sample_. The mean (blue line) and the 95% lower (orange line) and upper limits (green line) are plotted.

One problem with the comparison above is the difference in sampling points between the two subspecies. *V. v. nigrithorax* has 170 sampling points, whereas *V. v. variana* has only 20. If one sampled only 20 houses in Ottawa, the area enclosed by the convex hull polygon would underestimate the geographic distribution of Ottawa residents far more than an equivalent polygon from 2000 houses. This effect can be visualized if we subsample the 170 sample points for *V. v. nigrithorax* (Figure 6). When the number of sample points increases, the area enclosed by the resulting convex hull polygon also increases. For this reason, it is not fair to compare the area enclosed by 170 sampling points for *V. v. nigrithorax* with an area enclosed by only 20 points *V. v. variana*.

Figure 6 points to an approach for a fair comparison. When taking a subsample of 20 points from the 170 points for *V. v. nigrithorax*, the area calculated from this subsample is comparable to that from the 20 points for *V. v. variana.* The average area of repeated subsamples is 63.0385, with the 95% confidence interval of (57.3055, 304.4059). Because the calculated area for *V. v. variana* is only 47.1508, lower than the 95% lower limit, we may conclude that the distribution area of *V. v. nigrithorax* is indeed wider than that of *V. v. variana.* I believe that this conceptual framework can be used to compare invasiveness between any pair of sister lineages.

**Figure 7:**
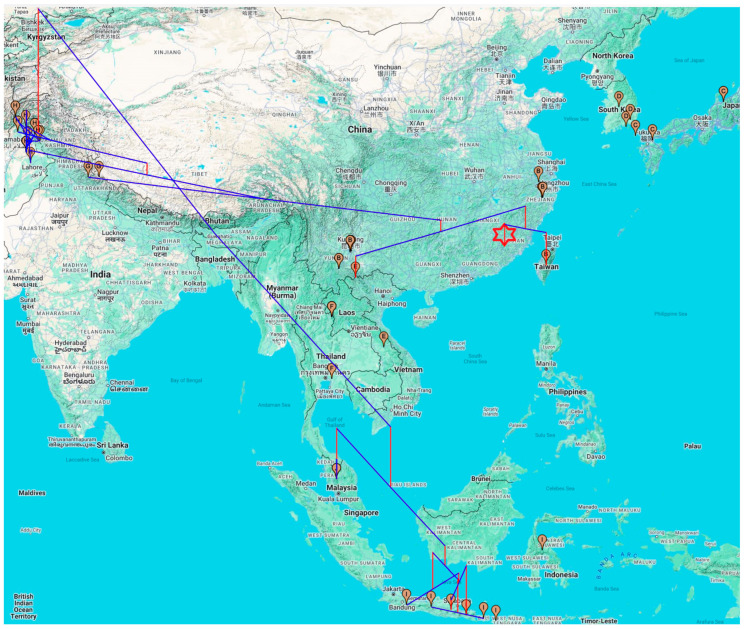
Geophylogeny of *V. velutina,* with specimens of the same country sharing the same letter in the map marker. The entire blue-shaded clade in Figure 5 was represented by a single red six-point star. All map markers not linked by the geophylogeny belong to this clade with a wide geographic distribution, which includes India, Indonesia, Vietnam, Laos, Thailand, China, South Korea, and Japan. The red vertical lines are proportional to branch lengths of the phylogenetic tree. Specimens connected by blue lines without any vertical red lines have branch lengths of 0, i.e., genetically identical. Locations were labelled with both English and the local official language.

The phylogenetic tree of *V. velutina* (Figure 5) is generally consistent with other studies. It was previously reported that *V. velutina* first diverged into two clades: an Indonesian–Malaysian clade and an Asian Continent Clade [43,67]. The latter lineage and subsequent derivatives colonizing Pakistan and India are generally sedentary, but a new invasive lineage (shaded in blue, Figure 5) arose, moved east, and colonized most of Southeast Asia. This is better visualized with a geophylogeny (Figure 7). The first split is between the Indonesia/Malaysian clade and the continental clade. The second split is between the sedentary India/Pakistan clade and an invasive clade that expanded not only to Southeast Asia, including Japan and South Korea but also to Europe (Figure 7). The general phylogeographic pattern in Figure 7 suggests allopatric speciation as the main speciation mechanism because, except for the recently derived invasive *V. velutina nigrithorax,* all other subspecies are generally separated geographically.

Contrasting the geographic points in Figure 4 and Figure 7, one would immediately notice the scarcity of points in Figure 7, i.e., many *V. velutina* populations are still not represented by molecular sequences. The scarcity of sequences would cause difficulties in tracing the source population. Two conditions are required to trace an invasive individual to its source population, i.e., populations should be genetically distinct and should be genetically characterized. If the two sequences from Jiangsu and Zhejiang in China (JQ780451 and JQ780453, colored blue in Figure 5) were not sampled, we would have to infer that the source population is represented by the three sequences colored in green (Figure 5). These three sequences were all sampled from Yunnan in the Southwest of China, thousands of kilometers from Jiangsu and Zhejiang.

When invasive individuals of *V. mandarinia* were caught in Washington State and British Columbia, they were found to be genetically nearly identical to specimens sampled in South Korea and Japan, respectively, and the source populations were therefore inferred to be somewhere in South Korea and Japan [16,33]. However, the populations along the eastern coast of China were poorly sampled. It is possible that the invasive individuals are genetically more similar to those *V. mandarinia* populations on the eastern coast of China. I highlight this in the hope that more targeted samples will be sequenced in the future.

## 4. Discussion

### 4.1. Species Invasion as a Natural Experiment Opens a Window into a Hidden Part of Nature

Invasive species are interesting mainly because of three consequences [68]. The first is the ecological and economic consequence mediated by the damage caused by the invading species on the fauna and flora, as well as threats to human health. *Vespa velutina* is a mass murderer of honeybees and causes significant damage to apiculture not only in Asia but more so in Europe [11,12,47,48,69,70]. *A. mellifera* has come in contact with hornets in two ways. The first is through the introduction *A. mellifera* into Japan and South Korea, where hornets exist either natively or through accidental introduction [43]. The second is through the accidental introduction of Asian hornets into Europe. If different honeybee colonies in Europe exhibit anti-hornet behaviors with different efficiency, then one should be able to observe the effect of selection mediated by the hornet predation on honeybee evolution, i.e., those with more efficient anti-hornet behaviors would be favored by the selection. It is also possible that the invasion may result in the extinction of specialized lineages representing thousands of years of adaptive evolution, such as *A. mellifera ligustica* and *A. mellifera carnica.*

The invasion of *V. velutina* may also exert selection pressure on local competitors, e.g., *V. crabro* in Europe or the multiple hornet species in South Korea. This may lead to three possible outcomes. First, when the population size is small for both species, then they may both increase in population size with increasing availability of prey species and other food items. When the population size of *V. velutina* (N_Vv_) and that of *V. crabro* (N_Vc_) are both high, and if they share the same diet and nest site requirement and the same carrying capacity of (N_Vv_ + N_Vc_), then one would expect N_Vv_ to be negatively associated with N_Vc_. Specifically, one would expect a successful invasive species to displace the native with a nearly identical ecological niche. This has been observed in South Korea, where the invasive *V. velutina* displaces the native *V. simillima* [28]. However, there is no clear indication that the invasive *V. velutina* in Europe displaces the native European hornet, *V. crabro* [63], suggesting that *V. velutina* in Europe does not face a local competitor sharing a similar ecological niche. This interpretation is consistent with the instantaneous rate of growth (*r*, Figure 3). The invasive individuals in Europe and South Korea should have similar biological features. However, *r* is much greater for *V. velutina* in Europe (with little competition) than in South Korea (with strong competition) (Figure 3). The second possible outcome of competition between an invasive species and its native competitor is that the two may be expected to differ in seasonal activities or daily rhythms, and they may diverge in nest sites and diet. The third possible outcome is that they may exhibit differential habitat preferences. In addition, the invasive *V. velutina* also provides new food for local predators such as the European honey buzzard, *Pernis apivorus* [30] and the European bee-eater, *Merops apiaster* [31].

The direct effect of *V. velutina* on human health appears minor. While the related Asian giant hornet, *V. mandarinia*, was known to attack humans and can kill a victim with an average of only 59 stings [71], *V. velutina* has not been a threat to human health. In France, only one severe case of envenomation was attributed to *V. velutina*, when a victim was stung 12 times on the head [72].

The second consequence of an invasive species is the reduction in genetic variation through the founder effect [73,74,75]. As Wright (1942) envisioned, a small population made of one or a few foundresses would facilitate the fixation of non-adaptive alleles or allele combinations. In most cases, the few foundresses and their descendants may just go extinct, leading to failed invasions. As seen in Figure 1, the observations of *V. velutina* in 1915, 1923, and 1980 likely represent failed invasions. However, if the few foundresses managed to generate a viable population, then any existing genetic variation or new mutations would evolve in a unique genetic background independent of the source population in a new habitat, potentially with a selection regime different from that of the source population. This may lead to a new evolutionary trajectory and facilitate speciation.

**Figure 8:**
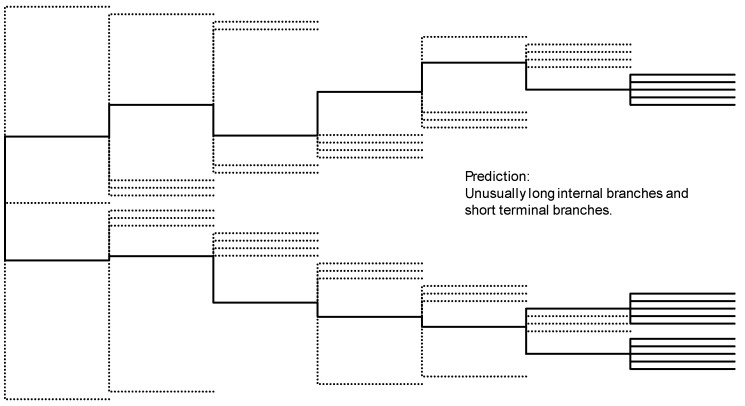
Phylogenetic consequence of inter-colony selection. Solid lines are surviving lineages. Dashed lines indicate extinct foundress lineages.

The source population may generate many invasive foundresses to explore new habitats. Some of these foundresses may carry many deleterious recessive alleles, and their offspring would suffer from inbreeding depression and go extinct, whereas some other foundresses may harbor few deleterious recessives and succeed in colonizing new habitats [76]. Population bottlenecks appeared responsible for purging off highly deleterious recessive alleles in the alpine ibex [77] and Iberian lynx [78], thereby creating a colony fitter than other colonies harboring more deleterious mutations. This mechanism also works in yeast species [79,80]. This inter-colony selection is depicted in Figure 8. A colony that has purged off most of the deleterious recessive mutations can spread and replace colonies with more deleterious recessive mutations. Such a successful colony would generate successful invasive foundresses because their descendants will have a better chance of escaping inbreeding depression when a few foundresses or just a single foundress moves into a new habitat with all genetic variation limited within her fertilized eggs. In this context, one may expect a successful invasive species should have low instead of high genetic variation, i.e., the descendants should distribute widely but with little genetic variation. *V. v. nigrithorax* fits these two criteria, as one can see in Figure 4 and Figure 7. This inter-colony selection would explain the observed paradox of successful colonizers carrying only a small fraction of the genetic variation present in the source population [81].

The third consequence of invasive species is the alteration of established biogeographic patterns mediated by human-facilitated dispersal. For example, the native distribution of *V. mandarinia* is in Asian countries and the Russian Far East [21,82,83] and is unlikely to disperse from Asia to North America by natural means. However, globalization and modern cargo transportation would make this possible, and multiple incidents of *V. mandarinia* queens found in British Columbia in Canada and Washington State in the USA have been documented and traced to source populations in Asia [4,16,33]. Vespine hornets appear to be highly capable of survival and reproduction in new environments and include multiple invasive species [2,3].

### 4.2. A Proposal for an Index of Invasiveness

There has been no explicit index of invasiveness, but my results suggest the conceptual framework in which such an index can be formulated. Designating the distribution area of a taxon as *A* and the divergence time of the taxon as *T*, the index of invasiveness (*I_inv_*) can be expressed as

(2) $$I_{inv}=\frac{A}{T}$$

The area *A* could be measured by the convex hull method that I have used in the paper, and *T* can be from phylogenetic dating. For taxa that evolve with a roughly constant rate, then *T* can be operationally replaced by branch lengths for comparison of *I_inv_* between sister lineages.

For *I_inv_* to be useful, the area *A* in Equation (1) needs to be estimated without bias. If Species X is more extensively studied than Species Y, with 10,000 sampled specimens for X but only 20 for Y, then the estimated *A* is likely greater for Species X than for Species Y, even if *A* is actually the same for both. Two approaches could be taken to alleviate this problem. The first is what I have used in the manuscript by randomly sampling 20 specimens repeatedly from the 10,000 specimens for Species X to calculate *A*_1_, *A*_2_, …, *A_n_*, and use the average of these *A_i_* values to compare against *A* estimated from the 20 specimens for Species Y. The second approach is to use ecological niche modeling (ENM) to obtain an area that can be potentially occupied by Species X and Y. This approach probably causes more problems than it is intended to alleviate. First, ENM will not generate informative output from an inadequately studied species with few sampling points. Second, species distribution depends on two factors: habitat suitability and dispersal capability. A suitable habitat may not be occupied by a species simply because the species has never had a chance to land in the habitat.

### 4.3. Pros and Cons of a Multi-Loci Phylogeny Versus a Single-Locus Phylogeny

Mitochondrial genomes are frequently used to reconstruct species-level phylogenies. The advantages of mitochondrial sequences include the following: (1) Rare participation in horizontal gene transfer, which is known to complicate phylogenetic reconstruction in bacterial species [84]; (2) Rare involvment in recombination; (3) Rare losses or gains; (4) High mutation rate in animal mitochondria, which is suitable for resolving species-level phylogeny; (5) Conserved structure so that one does not need to use complicated algorithms [85,86] for sequence alignment; (6) Abundance in cells; (7) Maternal inheritance that simplifies phylogenetic analysis. However, there are also several disadvantages inherent in mitochondrial sequences: (1) They are often too short with too few variable sites to provide sufficient power to resolve phylogenetic relationships; (2) Mitochondrial divergence may not reflect nuclear genome divergence, e.g., the genetic variation introduced by polyandrous mating in eusocial Hymenoptera [87] is not reflected in mitochondrial genomes; (3) Heteroplasmy [88,89,90], which can complicate phylogenetic interpretation; (4) Mitochondrial introgression, where the mitochondrial genome from one lineage gets incorporated into the gene pool of another, can obscure species relationships. For example, Hispanics have nuclear genomes that are typically European, but their mitochondrial genomes are from native women [91]. The interpretation is that European colonists wiped out most of the native men in Latin America but kept native women as their sexual partners, leading to the offspring having a native maternal mitochondrial genome but a European paternal nuclear genome. Such genetic introgression would also complicate genealogical relationships.

This paper used only mitochondrial sequences for phylogenetic and phylogeographic analyses. It is not clear if mitochondrial heteroplasmy or mitochondrial introgression occurs in *V. velutina.* However, mitochondrial heteroplasmy was documented in an Australian native bee species, *Amphylaeus morosus* [92], and mitochondrial introgression occurs between two mosquito species [93]. In this context, it is advisable to use multi-loci data instead of single-locus data for phylogenetic reconstruction. If one uses mitochondrial sequences only for phylogenetic analysis, one should at least use multiple sequences from each species or subspecies.

### 4.4. The Taxonomy of Vespa Velutina Lineages

*Vespa velutina* populations have been found in diverse habitats in Southeast Asia and have evolved different color forms and morphological characters. The taxonomy of *V. velutina* was traditionally based heavily on color forms, leading to the designation of several subspecies [22,94]. For example, *V. v. auraria* and *V. v. nigrithorax* were sympatric in northern India but maintained their distinctive color patterns [20,22,94], indicating reproductive isolation. This led to the elevation of *V. v. auraria* to the species level. However, intermediate color forms were subsequently observed [95,96], so the species designation of V. auraria was reverted back to subspecies. The distinct color form for *V. v. variana* [24], was sympatric with *V. v. nigrithorax* in northern Vietnam [24,95,96]. However, color patterns can be highly misleading as a taxonomic marker. The most dramatic example is perhaps the large ladybird beetle *Harmonia axyridis*, which had 105 named morphs and was classified into several genera [97,98]. However, detailed breeding and genetic analysis demonstrated that all color morphs can breed with each other and produce offspring with no detectable reduction in fitness. Furthermore, the dazzling array of color morphs is determined by a single locus with 12 different alleles [97,98]. Thus, all different color morphs belong to a single genetically coherent species. The recognition of the inadequacy of color patterns as a taxonomic marker has led to almost all subspecies in *V. velutina* being synonymized [83].

The taxonomy of *V. velutina* in the last few decades has relied on morphological differences other than color forms [95,96] or a combination of morphological and color variation [21,23]. However, while color differences may exaggerate the underlying genetic divergence, morphological similarity often hides genetic differences. A prime example involves the two morphologically nearly identical ‘races’ of fruit flies now known as *Drosophila persimilis* and *D. pseudoobscura* [99]. Not only could entomologists fail to tell them apart based on morphology, but fruit flies also could not distinguish between the two and readily mate with each other to produce infertile offspring. The two ‘races’ exhibited large-scale genetic differences, including many chromosome inversions [99].

Molecular sequence data have been the most reliable genealogical markers, and it is generally accepted that taxonomy should reflect phylogeny established by molecular phylogenetics. The total evidence incorporating morphological and molecular data have recently been used for reconstructing phylogenetic relationships of hornet species [46], specifically of *V. velutina* [43]. However, the resulting phylogeny did not provide clear resolution within *V. velutina.* For this reason, the subspecies designation in this manuscript is tentative.

The phylogenetic relationship (Figure 5) and the phylogeographic pattern (Figure 7) showed a clear split of the ancestral *V. velutina* into two lineages. One lineage is distributed in Indonesia/Malaysia, and the other colonizes the Asian continent. The Asian continental lineage includes several sedentary clades inhabiting India and Pakistan and an invasive lineage colonizing Southeast Asia. This latter lineage includes *V. v. variana* and *V. v. nigrithorax*, which invaded France.

### 4.5. Invasive Species and Ecological Niche

An ecological niche of an evolutionary lineage such as a species is a loaded concept that can take different meanings [100,101]. Conceptually, the niche includes two sets of specifications. The first set specifies the (biotic and abiotic) environmental requirements for the long-term propagation of the lineage. The second set specifies the effect of the species on the environment. An invasive species needs to find its ecological niche in a new location that satisfies its long-term propagation, and it typically alters the environment of those species that have not been exposed to the new invader before.

Biologists have made progress with the first set of specifications. The environmental requirement for long-term propagation has been characterized for many microorganisms in the form of minimum culture medium. Phylogenetically similar species tend to have similar ecological requirements relative to phylogenetically distant species. The recent development of ecological niche modeling (ENM) represents a more general approach to characterizing the environmental requirement of different species [102]. Such modeling typically integrates biotic factors such as prey, predator, competitor, etc., with abiotic factors such as temperature, humidity, vegetation, soil, etc.

There has been little methodological advance in the second set of specifications, i.e., the effect of the species on the environment. The commonly used approach is the keystone species analysis initiated by Paine [103], which shows that the removal of predatory sea stars (*Pisaster ochraceus*) caused dramatic changes in biodiversity in the rocky intertidal. It is also interesting to note that the COVID-19 pandemic resulted in a substantial reduction of other respiratory pathogens and their associated respiratory diseases [104]. In this context, SARS-CoV-2 is also a keystone species in the community of respiratory pathogens.

How would an invasive species such as *V. velutina* affect the European habitat that it has successfully colonized? It could reduce the population size of honeybees by preying upon them, or of *V. crabro* by competing with them for food and nest sites. It could serve as new prey items for European honey buzzard [30] and the European bee-eater, *Merops apiaster* [31], thereby increasing their population size. Such interactions have not yet been empirically studied.

ENM serves not only to approximate the ecological niche of a species and to predict the species distribution but also to pave the way for studying species interactions and how such interactions would also alter the environment. Two species with similar ecological requirements as predicted from ENM are expected to competitors. The density of such competing species should be negatively correlated, which appears to be the case between *V. velutina* and *V. simillima* [28]. Conversely, two species exhibiting competitive exclusion are considered to have similar niche requirements. Phylogenetic similarity might also serve as a proxy for niche overlap. There should be a quantitative measure of niche overlap to facilitate the study of the effect of invasive species on the fauna and flora of the newly colonized location.

## 5. Conclusions

Existing biodiversity databases such as the Barcode of Life Data System, Global Biodiversity Information Facility, and GenBank can be used to address many questions concerning invasive species. Phylogeographic software such as PGT takes advantage of such publicly available data to generate geophylogenies to visualize the distribution of genetic variation over time and space, estimate population growth parameters such as intrinsic rate of growth of the invasive population, infer the time of the last successful invasion, and characterize species invasiveness by computing biogeographic distribution areas. These analyses were applied to the study of the invasive yellow-legged Asian hornet, *V. velutina.* The last successful colonization of Europe and South Korea occurred in late May and early October 2002, respectively, by different source populations in Southeast China. The intrinsic rate of population growth is greater in Europe than in South Korea, which is consistent with the interpretation of little competition in France and strong competition from local hornet species in South Korea. The invasive potential of *V. velutina* is higher than its sister lineages because it has expanded and occupied a larger area than its sister lineages. The ancestor of *V. velutina* split into two lineages, one was found in Indonesia/Malaysia and the other colonized the Asian continent. The second lineage split into a sedentary clade inhabiting India and Pakistan, and the other invasive one colonized much of Southeast Asia. This latter lineage gave rise to the subspecies *V. v. nigrithorax* that invaded France, South Korea, and Japan.

## Data Availability

Data are contained within the article.

## Supplementary Materials

The following supporting information can be downloaded at: https://www.mdpi.com/article/10.3390/life14101293/s1. It contains two files: (1) Final_Vespa_velutina_Phylogeny.pptx which is the uncollapsed version of the tree in Figure 5, and (2) Table S1: Sequence_Information.xlsx which contains sequence ID, latitude and longitude.
