# Supplementary material for: Phylogeographic Analysis for Understanding Origin, Speciation, and Biogeographic Expansion of Invasive Asian Hornet, Vespa velutina Lepeletier, 1836 (Hymenoptera, Vespidae)
Source: Life (Basel). 2024 Oct 12;14(10):1293. doi: 10.3390/life14101293 (PMC11509734; doi:10.3390/life14101293)
Supplement: Supplementary file 1 [file life-14-01293-s001.zip › Final_Vespa_velutina_Phylogeny.pptx]

## Slide 1
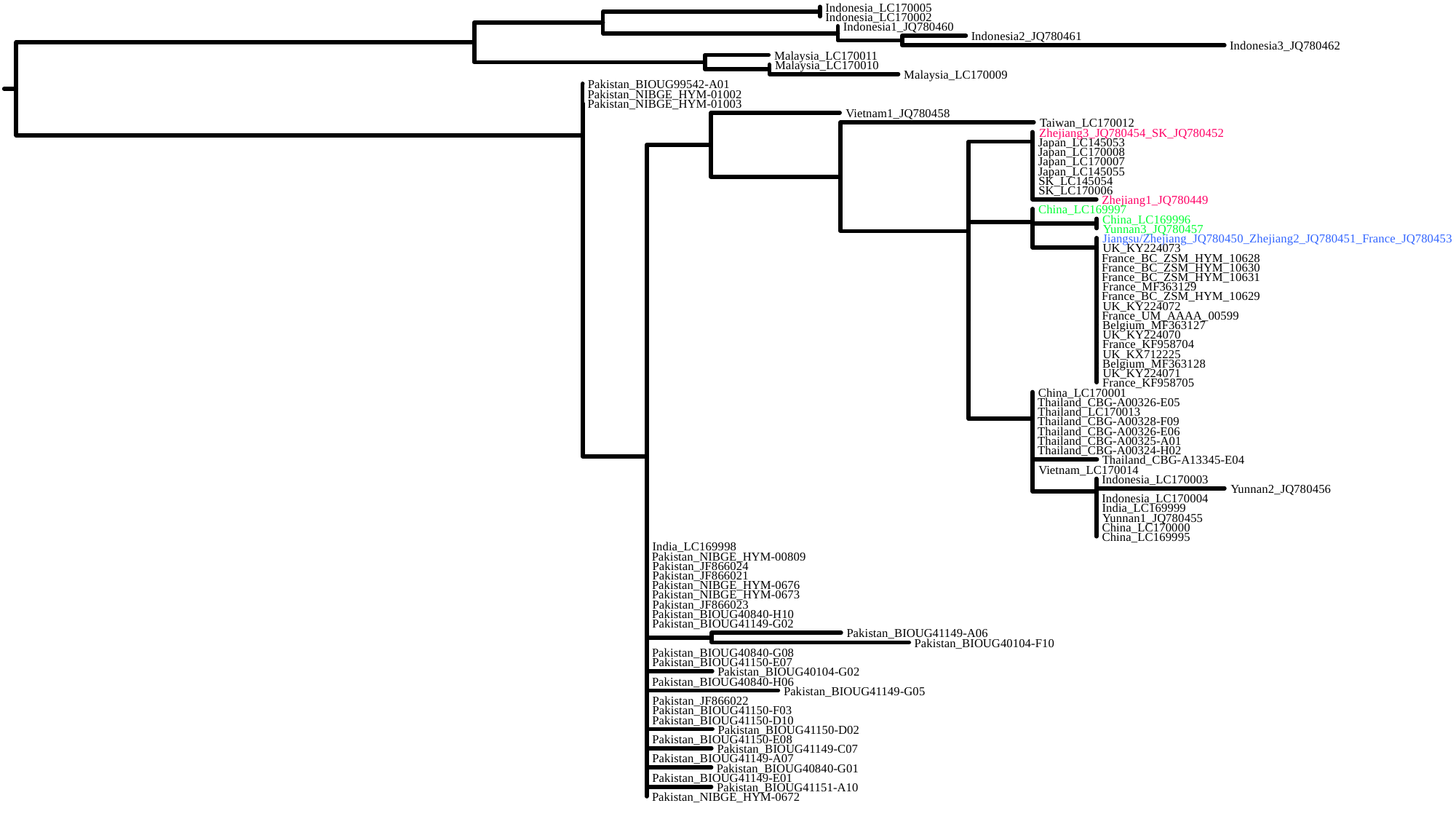

Indonesia_LC170005
Indonesia_LC170002
Vietnam1_JQ780458
Indonesia1_JQ780460
Indonesia2_JQ780461
Indonesia3_JQ780462
Malaysia_LC170011
Malaysia_LC170010
Malaysia_LC170009
Pakistan_BIOUG99542-A01
Pakistan_NIBGE_HYM-01002
Pakistan_NIBGE_HYM-01003
Taiwan_LC170012
Zhejiang3_JQ780454_SK_JQ780452
Japan_LC145053
Japan_LC170008
Japan_LC170007
Japan_LC145055
SK_LC145054
SK_LC170006
Zhejiang1_JQ780449
China_LC169997
China_LC169996
Yunnan3_JQ780457
Jiangsu/Zhejiang_JQ780450_Zhejiang2_JQ780451_France_JQ780453
UK_KY224073
France_BC_ZSM_HYM_10628
France_BC_ZSM_HYM_10630
France_BC_ZSM_HYM_10631
France_MF363129
France_BC_ZSM_HYM_10629
UK_KY224072
France_UM_AAAA_00599
Belgium_MF363127
UK_KY224070
France_KF958704
UK_KX712225
Belgium_MF363128
UK_KY224071
France_KF958705
China_LC170001
Thailand_CBG-A00326-E05
Thailand_LC170013
Thailand_CBG-A00328-F09
Thailand_CBG-A00326-E06
Thailand_CBG-A00325-A01
Thailand_CBG-A00324-H02
Thailand_CBG-A13345-E04
Vietnam_LC170014
Indonesia_LC170003
Yunnan2_JQ780456
Indonesia_LC170004
India_LC169999
Yunnan1_JQ780455
China_LC170000
China_LC169995
India_LC169998
Pakistan_NIBGE_HYM-00809
Pakistan_JF866024
Pakistan_JF866021
Pakistan_NIBGE_HYM-0676
Pakistan_NIBGE_HYM-0673
Pakistan_JF866023
Pakistan_BIOUG40840-H10
Pakistan_BIOUG41149-G02
Pakistan_BIOUG41149-A06
Pakistan_BIOUG40104-F10
Pakistan_BIOUG40840-G08
Pakistan_BIOUG41150-E07
Pakistan_BIOUG40104-G02
Pakistan_BIOUG40840-H06
Pakistan_BIOUG41149-G05
Pakistan_JF866022
Pakistan_BIOUG41150-F03
Pakistan_BIOUG41150-D10
Pakistan_BIOUG41150-D02
Pakistan_BIOUG41150-E08
Pakistan_BIOUG41149-C07
Pakistan_BIOUG41149-A07
Pakistan_BIOUG40840-G01
Pakistan_BIOUG41149-E01
Pakistan_BIOUG41151-A10
Pakistan_NIBGE_HYM-0672
